# Supplementary material for: Virus detection via programmable Type III-A CRISPR-Cas systems
Source: Nat Commun. 2021 Sep 27;12:5653. doi: 10.1038/s41467-021-25977-7 (PMC8476571; doi:10.1038/s41467-021-25977-7)
Supplement: Supplementary file 1 — Supplementary Information [file 41467_2021_25977_MOESM1_ESM.pdf]

SUPPLEMENTARY INFORMATION

Virus Detection via Programmable Type III-A CRISPR-Cas Systems

Sagar Sridhara[1†], Hemant N Goswami[1†], Charlisa Whyms[2], Jonathan H Dennis[3], & Hong Li[1,2*]

[1]Institute of Molecular Biophysics, Florida State University, Tallahassee, FL 32306, USA.

[2]Department of Chemistry and Biochemistry, Florida State University, Tallahassee, FL 32306, USA.

[3]Department of Biological Science, Florida State University, Tallahassee, FL 32306, USA.

[†]authors contributed equally.

[*]Corresponding author: hong.li@fsu.edu

Supplementary Table 1: Amino acid sequences of *Lactococcus lactis* CRISPR-Cas (Csm) system. Related to Figures 1, 2 and 3.

Subunit	Amino acid sequence
Cas10/Csm1	MDKINLVCGSLLHDIGKIIYRGTSERAKHSKLGGDFIKSFEQFRNTELTDCIRYHHAQEITSVKSNKEKN<br>SLFYITYIADNISSGMDRRKDLEEGAEGFNWDKKVALGSVFNVLNEKEKGRQNYSYPFVARTRIKEEPLN<br>FPTATQNQYTTSYYDGLITDMKTILQRLKPDKEHINSLLQMMESLWSYVPSSTDKNQLVDISLYDHSRTT<br>AAIASAIYDYFQAENITDYQKELFDYNATEFYDKNAFLMMNFDMSGVQNFIYNISGSKALKSLRARSFYL<br>DMLLEYISDNLLEKLELSRANILYVGGGHAYLLLANTNKTKAILSDFEHDLKTWFLDKFKIDLYVAMAYT<br>EVSANDLMNHNGHYRDIYRRLSQKTSAKKANRYTAEEILNLNHQGTENARECRECKRSDLLIEEDDICEI<br>CDSLQKVSRDLTRENIFVIANEGVLDMPFGKKMSALSYSQADKLKKSNAEVQIYAKNISEIGQNLMTRID<br>MGDYTYRSDFHEMLEEVEVGINRLGVLRADVDNLGQAFINGIPDDYLSISRTATFSRAMSRFFKNYLNQL<br>LAEKSYKINVIYAGGDDLFMIGAWQDILDFSIVLKQKFADFTQNKLSISAGIGMFREKYPVARMASLTGD<br>LEDAAKDYKPDERAVQATKNAVTLFDATNVFSWDTLENDIFVKLDAITKNFEKLDETGKAFIYRLIDLLR<br>GVNENQQINIARLAYTLSRMEEKIGKTFAQELYNWANADRKTLIMALEIYILKTRER
Csm2-NHis	MGHHHHHHSGGTELKIGNEKVNSTNFGDFAEKAIRGINHKPFVNSKGGEQKITTSKIRGILELVNKVYNR<br>VINTNDVELSENILADIAYIKVKIAYESGREPVVKDFIQRTAFTAAITDVMNQRTRESFLLFARYVESLI<br>AYFKFYGGKD
Csm3	MKLVIEGTIVLKTGMHIGGSSDFSAIGAVDSPVVRDTLTRLPLIPGSSLKGKMRYLLAKELNNGILLNEP<br>NNDQDEILRLFGSSEKDKIRRARLKFNDIKLSNLAELETFNVSSTEVKFENTINRKTAVANPRQIERVIA<br>GSKFDFEIFYNLDDIKEVEKDFENIKQGFDLLEFDYLGGHGTRGSGRIAFENLSVITAVGNFEKINTLNE<br>ILGA
Csm4	MKIIKLYFESPVHFGEKRLSESKITFSADTLFSALMIEAVGLGKEDEFYQLASNNLVKFSDAFPFIDQYY<br>YIPKPMFNLKLEKEDENPSKAFKKLLYVPIDSLEDYLSGGLDAYFERESFNLGKLALSEKVQQHDFKDSE<br>PYNVGTFTFKENTGLYVLIEQTHPLLEELLENLQYSGIGGKRNSGYGKFKFEILEDSDIEDLFSAKGNRK<br>ILLSGALPKDAELEQALKNASYLLERRGGFVQSDTYATNLVKKQDLYVFKSGSTFENSFDGDIYQVGKKG<br>NHPVYKYAKSFFLEVSV
Csm5	MKKTYRVTLTALGPIFIGGGEKLKKYEYIFDKQKKVAHMIDHTKFTKYLLEKNLLDDFTSRVNSHFDLYD<br>YLVNKKGIVFMPLVKYSVPVAQFRTEVKNRFGKPISSPPMNDLNTFVKDAFGRPYIPGSSLKGALRTAIL<br>NDLKEDTKENEVFAHLQVSDSETIDLENLKVYQKVDYSKTAKPLPLYRECLKPNTEITFTVSFDDEYLTL<br>KKIQNALHKTYQHYYIKWLKGGKVGETLIKGVYDSHADELKKNTFALDQPSQNQGEIIYIGGGAGFVSKT<br>LHYKSKNRDQARNDSFDILKQLFRTTYSKMRSVPDNVPVALKLAVETKTFNGRVTGKHYLEMGKARIKLE<br>ELK

Csm6	MKILISAVGDTDPIRNFHDGPLLHIVRVYRPEKIVLVHSERSLTKHDKLVKALKSIKDYSPEIIQDGVVL<br>PDAQVAIFDEMYDTVSSIVKKYISDDEIILNISSATPQIISAMFAVNRISDFNVTAVQVKTPQHKSNEGL<br>RHDNQEDIDKLIETNLDNQSDYENRTLADTGMKFSQDLTKRNLKALIDNYDYQGALELLKKQKSFSNIKE<br>LRKKLTEISDTIKIQGMPDKIVKSKLSNQAKSALNSYLNIDRNHKQGNIAEVLIRVKSLVEFILEDYLNN<br>HFLDVITYKDGKPFLNASKYPEILKKFQEDAEMRGKEYHSGYLSLPAYIGILKFFEPNHDLLKHIYKIQE<br>INQDRNKVAHSLQAFDRKNLKKVSSAVFASKQILLASFDIDNHWFSFYEDLNQEIKKLL
Cas6	MIVKLRYKINLPNSLRTQNIGSTLHGVLMELLPSELVEHLHNLSYNPFRQRLIFEKELVIWEIVGLHKMV<br>SEELLKLENLREITIKRAQKTVSLSLLSKDAIAVDDLVKKEMGREIDSRIISLKFTSPTSFKANGHYDIF<br>PDIRKIFRSLMMNFDFFSETTKIYDYEVLSYIEENVHIVSYKLMTKNFHLEKIKVKGFQGDMTLKVTGAE<br>QFVKLVLLMIKYATFAGIGMKTSLGMGGVSINERHYLR

Supplementary Table 2: Sequences of crRNAs used in this study. The 37mer crRNA sequences are shown highlighting reprogrammable 29mer protospacer region (bold). The 8-nt crRNA 5'-handle sequence (small letters) originates from the repeat sequence processed by Cas6.

Name	Sequence	Target	Figure
Model LlCsm crRNA	5′ acgagaac**AUACGUUCUUUGAACCAAGCUUCAACUCC** 3′	Model target	Fig 1, Fig. S1
LlCsm_S_0 crRNA	5′ acgagaac**GCAGCACCAGCUGUCCAACCUGAAGAAGA** 3′	S0_CTR, S_IVT_RNA, COVID patient extracts	Fig 2, 3, Fig. S2, S3
LlCsm_S_7 crRNA	5′ acgagaac**UGCAGGGACAUAAGUCACAUGCAAGAAGA** 3′	S_IVT_RNA, COVID patient extracts	Fig. 2, Fig. S2
LlCsm_S_8 crRNA	5′ acgagaac**GCAACCUCAUUGAGGCGGUCAAUUUCUUU** 3′	S_IVT_RNA, COVID patient extracts	Fig. 2, Fig. S2

Supplementary Table 3: Sequences of target RNAs in this study. The 37mer target sequences are shown highlighting 29mer region complementary to crRNA protospacer (bold). The corresponding 8-nt 3'-protospacer flanking sequence (3'-PFS) is shown in small letters. The 3822 bp long S_IVT_RNA sequence is shown highlighting the 3'-PFS (small letters/underline) and target RNA protospacer (in yellow/bold) for targets: S_8, S_7 and S_0.

Name	Sequence	Compatible crRNA	Figure
Model target	3′ acgagaac**UAUGCAAGAAACUUGGUUCGAAGUUGAGG** 5′	Standard LICsm crRNA	Fig. 1, Fig. S1
S_0_CTR	3′ acgagaac**CGUCGUGGUCGACAGGUUGGACUUCUUCU** 5′	LICsm_S_0 crRNA	Fig. S2
S_IVT_ RNA	3′<br>GUGUUCUUGUUGUCGGGAACUCUGUUGAUGUCGUUGACCAGUAUGUCGUUUCGUAUUAACAG<br>UGGUAAUGAUACCGUUAGUUCGGUCGAUAUUUUGGAUCGGUUUACAUGGUACCGGUAAAAUA<br>UAUGACGAGUAUGAAAGGUUCAAGAACCUCUAGCUACUCUCUAAGUAAAUuuaagaac**CGUU**<br>**GGAGUAACUCCGCCAGUUAAAGAAA**AACUUACAAAUGUUGACUUCGUAAUUACGGUCUCUAC<br>AGUGGAUUUAGUUGUAGACCACUACAUACUAAGAAUUUUAUAAAUAGAUUGAGGAGGAACUU<br>ACUCAGAUUAAGUCCAACGUUUCCUAGUAUUUGACACAACAACUGUUAAGGAUAAUGUUGUA<br>GUGUCAAUGGUCUGUGUUUACACAACAGACAUCAUUACUAAACACCAAGUAUUUUUAAGGAA<br>ACACAAUGUUUGGUCACACACGGUAAACUUUGUUUCUGUGGAAGUGCUCCUUUCACACGAAA<br>AGGUAGUACUGUUUACCGUCCUCGUCAACACUUCAAGaaaagaac**ACGUCCCUGUAUUCAGU**<br>**GUACGUUCUUCU**GAUGUGGUACUCCACGACUGACUCCCUUCCUGUAUUCUACUAUCGGGAAA<br>GGUGUUUUUAGUUGAGAAAAACUAACAGGUUCAUGUGUGAGACUGUAAAAUCAUCGUCGUUC<br>UAAUCGUCUUCGAGACUAAAGACGUCGAGAUUAAUUAACAACUCAGUGUAUACAGACGUUUG<br>AAACUUCAGACGGACACUAGUUGGAUAGUUAAACGUGAAGUCGGAGUUGAAACAGUUCUGCA<br>CUUUCCUAUAGUAAAUUUUGUGAACUUUAACGUGGUUUUAACCUCGAUUCAACAAAUUGUUC<br>GCACAAAUUUCGAACACGUAAAACCAACUGGUGUAGAACUUCAAAAGGUUCACGUGAACGAC<br>ACCUUCUUUCACUCAGAACUUAAAACGGUUAUCGUGAUAAUUUAACCAACCGUUAGUUAAAA<br>ACCAAGAGUAUCUCUUGUAAGACACAUUGAGGUUAUGGUAAUUUGGAUAUUCGGUAAACGUA<br>UCGUUUACCAUAAACAUUACGUCGUGGACGUGGUUUCCAGGUUGGUCUUCACUAACAUGGGC<br>GAUUGUCACGUCUUCACAUAACUCGUUAGUAAAGUAGACACUCGUUUCCACCGUUUUGUCAU<br>UCCGGCAAUUUGAAAACACGUGUUUACUCCAGAGAUCGUCGUUAUAGUGGUUCCGUUAGUGG<br>UAUAACAAACUACUUCGGUCGUAGACGUUCACAGUGAAACAACUUUUCAUCUAGAAGUUAUU<br>UACUGGAGAACGAACCAAAACUACCUAGACCAUUAUAAACACUUUUUAAUUUUGGUGGUUUU<br>AGAAAUUAACCACCACAAAACAUUUAAACAAACUGAACACGUUUUUGAAGAACCCACAAAAA<br>CAGAACAAGUUGUCGAUAAGGUCAAUUUCGUGCCAAAUUAACACAUGUUUUUGACGGUAUAA<br>CGUUGUUUUCUAACGACGUAAGUCAACUUAGUGGUGUUUACAUGUAACAUGUUAGAUGACUA<br>CAGAACCAGUAUCUGUGACCAUCUUAAAGACACCAUUGUGAUUAUCAUUUUAAACACCCAUA<br>CCGUUAUCUCAAUAAUCUCAUUCGUUGACUUAAAAGACGUGGUUCACUGUAUCACAUCCGUU	LICsm_S_8, LICsm_S_7, LICsm_S_0 crRNA	Fig. 2 & 3, Fig. S2 & S3

	ACUACCUAACUGAUCGAUGUGAUGCACGGGCGGCUCCUCUUAAUCAGACUCAGACUAUUGAU<br>CGCGUAUAUGGACGUGGUUACCCAUACAGUGUGAGUAUACUCAACAACUGUACAAGUCGGGG<br>AUAAUUUGUCGGACGUGCACAAACUUUUUGUAAUCUUGGACAUCUUAUUUGUGCGGUUCAUC<br>CUCAUUCAACUAGACGUACUUAUCGUUGUCCCUGAAGACACGUCAAUUGUAGGACUAUUUCU<br>UGUCGUUGGACCAAUCUUCAUAAACAAGGACCACAAUAUUGUGACUGUGGUGGUUUUCUUGU<br>ACCACAUUACAGUUCUUAGAGUUCACAGACACCUAGUGCCUGUCGUAGUCAUCACAGUCGUU<br>ACAGAGACGGUUUAACAACCUUUCCGUCUUUGAAAAACAAUCUGAGUCAUUCUUGUGGACAC<br>GGACAAUUUGGUAACUUCAACUUUAACUGUGUAAACAAAAAUUGGUUUAAUCAUCUGAAAAA<br>UCCAGGUGUUUGUCAACGACCACGUACAUCUUCAAGUUUUCUUUCAUGAUGAUGAGACAUAC<br>CAACCAUUGGUUGUGGUAAUCACCCAACCUUUGGUAUACUAACAUUUCCUUUCAUUGUUAAU<br>UUUGGAAGUUGUGGUAAUGUUCCACACGAUGGCCGGACUAUCUAAAGUCAACUUUAUAGAGA<br>GAGUUUUCCAAACUCUAAUCUGAAGGAUUUGUUAGAUAUGUCCAUUAAUAUUAAUGGUGGUU<br>GGAAUCUUAGUUCUAACAAUCUUAAGGUUCGAUAUUGCGUCGGACAUUUUAGUAGACCAUUA<br>AAUAUUAAUAUUAGUCGUUAGAAAGGUCAAACGGGACCUCGCUAAACAGACUGAAGUAGUGG<br>AGAUUAAUGUUUACUUAGACGUAUCUGUAAUCAUUUCGUCUCUAGUAAAUUAAAUCAUCCUC<br>UGUGAGGUAUUGUGAAUUUUCACCUUUUACUACGCCUUAAUAUAUCCUGUCUUAUUAGUCGU<br>UGUGUCAACGACUAAGAGAAGGACAAGGUUCGUAUUUGUCUACGUUUAGACCACCGCAAUUU<br>UUGAAGUGGUUUUCCCGUGUUCAAACAUUAUAAUCCUUUAGAUUGUUAUCUAAGACAACCAA<br>CCUGAGAUUUCAAUCUUCAAACUAUCUAAGGAAAAAGAUGUCACUUCCUAAAGUUGCAUGUG<br>AAACAAAGACUCUCUCCCAGUUCACGUGUCAGAUGUCGUAGACAUUACCAAGGUAAAAGUAA<br>UAUAAAAUUAUCUUUUCAGGAUCCAACUUCUAUUGGGUGUAuuauucga**CGUCGUGGUCGAC**<br>**AGGUUGGACUUCUUCU**UAGUGGUCCUCAGUUUAUUGAAGAUACAUUUCGUUCAUUUCAAACU<br>UUGGAUCACUACAAUUAUGGAUAACCGUUUAGAUGGUUACCAAGAUUUCGGCUUUUUGGGAC<br>UCCCUCUAGUGCGUGAUUUAAUUAUCCGCACACGAAUCUUAUAUAAAAUUUUAUUGGUAGUU<br>AUAAGAAUUUGUGUUUAAGGGAUUCUAAAAACUUUAAUGGGACAAAAGGAAGUUCCAGGUAU<br>UCUUUUCCGACUCUCUGUAUAAGUUUUCACGUUAAUAAGCGUGAUCUUAUUUGAGACUUGAG<br>UGAAAGGUAGGUUGAAAACAACAAAAACACCAUUAUUUGUGGGUUUUUACCUAGUAAUGUUU<br>UAACUUUAAGUGUCUGAAAUUAUUGUUGUAAUCAUCGCAAUAAUUGUUAUUCAUCCCUGACC<br>CAGAAGCUUAGAUUUCAUCAUGGUUUUUAGGUCGGAGAAUAAUACAAUCUGAAGAGUCACCU<br>UCGUUUUAUUUGUGGUAGUAAUUUACCAUCCUGUCCCAAUAGUUUGGAGAAUCAUGGUAACC<br>AGGGUCUCUGUACAUAUCGUACCUUGGUUCAUUGUAACCUUUUCUUUCCAUUCUUGUUCAGG<br>ACUCAACUUACAUUUUGACUCCUAGACUUUUGAAACAGUCCCAUUAUUUGUGGUGCACACUU<br>UCUUAAUCACAUACGUCCCCCAUUAACUCAAGACCAACAUUCUAAUUGUGUGACUGAUCUCU<br>GAUCACCGUUAUUUUGUUCUUUUUGUUUGUAUCUUCUCAGAGGAAGAAUUUCAAUUUGUUUU<br>AAUAAAGAUCUCCCCUUAACAAUAGGCGAGUGUUAAGGGG 5’		

Supplementary Table 4: Sequences of fluorescent probes used in this study. The oligo sequences of fluorophore-quencher system are defined with Integrated DNA Technologies (IDT) nomenclature to facilitate ordering. `/5Alex594N/` is a mod code for 5' Alexa Fluor® 594 (NHS Ester) dye with Absorbance max at 584 nm and Emission max at 616 nm. `/3IAbRQSp/`is a mod code for 3' Iowa Black® RQ quencher with Absorbance max at 667 nm. `/56-FAM/` is a mod code for 5' 6-FAM (Fluorescein) fluorophore with Absorbance max at 495 nm and Emission max at 520 nm. `/3IABkFQ/`is a mod code for 3' Iowa Black® FQ quencher with Absorbance max at 531 nm.

Name	Sequence	Fluorophore	Excitation/Emission	Source
DNA-Alexa	/5Alex594N/TTATTATT/3IAbRQSp/	5' Alexa Fluor® 594	570/630 nm	IDT
DNA-FAM	/56-FAM/TTATTATT/3IABkFQ/	5' Fluorescein	480/530 nm	IDT
RNA-FAM	/56-FAM/rArArArArA/3IABkFQ/	5' Fluorescein	480/530 nm	IDT

Supplementary Table 5: Compilation of coding and non-coding strands of T7 transcription templates and RT-RPA primers used in the study. Related to Figures 1 & 3. The double-stranded DNA templates for T7-MORIARTY/RT-RPA-T7-MORIARTY were prepared by annealing single-stranded, complementary forward and reverse primers (Eurofins Genomics). The T7 promoter region in the forward primer and its complementary sequence in the reverse primer are highlighted by yellow. The region corresponding to 3'-PFS of transcript is underlined. Mutated nucleotides for specificity studies are highlighted in cyan. The italicized region represents primer segment annealing to the target.

DNA	Sequence	Source
Model target	5′ TAA TAC GAC TCA CTA TAG GAG TTGAAG CTT GGT TCA AAG AAC GTA TCAAGA GCA 3′<br>5′ TGC TCT TGA TAC GTT CTT TGA ACCAAG CTT CAA CTC CTA TAG TGA GTCGTA TTA 3′	Eurofins Genomics
S_0_CTR	5′ TAA TAC GAC TCA CTA TAG TCT TCT TCA GGT TGG ACA GCT GGT GCT GCAGCTT ATT 3′<br>5′ AAT AAG CTG CAG CAC CAG CTG TCC AAC CTG AAG AAG ACT ATA GTG AGT CGT ATT A 3′	Eurofins Genomics
S_0_CTR_noT7	5′ G TCT TCT TCA GGT TGG ACA GCT GGT GCT GC AGCTT ATT 3′<br>5′ AAT AAG CTG CAG CAC CAG CTG TCC AAC CTG AAG AAG ACT3′	Eurofins Genomics
S_0_CTR_mm+1	5′ TAA TAC GAC TCA CTA TAG TCT TCT TCA GGT TGG ACA GCT GGT GCT GGAGCTT ATT 3′<br>5′ AAT AAG CTC CAG CAC CAG CTG TCC AAC CTG AAG AAG ACT ATA GTG AGT CGT ATT A 3′	
S_0_CTR_mm+5	5′ TAA TAC GAC TCA CTA TAG TCT TCT TCA GGT TGG ACA GCT GGT CCT GCAGCTT ATT 3′<br>5′ AAT AAG CTG CAG GAC CAG CTG TCC AAC CTG AAG AAG ACT ATA GTG AGT CGT ATT A 3′	Eurofins Genomics
S_0_CTR_mm+1_mm+5	5′ TAA TAC GAC TCA CTA TAG TCT TCT TCA GGT TGG ACA GCT GGT CCT GGAGCTT ATT 3′<br>5′ AAT AAG CTC CAG GAC CAG CTG TCC AAC CTG AAG AAG ACT ATA GTG AGT CGT ATT A 3′	Eurofins Genomics
S_0_RPA_F (forward primer)	5′-GAAATTAATACGACTCACTATAGGGAGGTTTCAAACTTTACTTGCTTTACATAGA-3′	Eurofins Genomics
S_0_RPA_R (reverse primer)	5′-TCCTAGGTTGAAGATAACCCACATAATAAG-3′	Eurofins Genomics
S_T7minus_F (forward primer)	5′-AGGTTTCAAACTTTACTTGCTTTACATAGAAGTTATTTGACTCC-3′	Eurofins Genomics

Supplementary Table 6: The target DNA sequences of Nucleocapsid (N), Envelope (E) genes of SARS-CoV-2 and human RNase P control used in qPCR validation.

	**Measurand**	**DNA Sequence (5' - 3')**
**Target 1**	SARS-CoV-2 Nucleocapsid; 28287 - 28306 (NC_045512)	GAC CCC AAA ATC AGC GAA AT
	SARS-CoV-2 Nucleocapsid; 28335 - 28358 (NC_045512)	TCT GGT TAC TGC CAG TTG AAT CTG
	SARS-CoV-2 Nucleocapsid; 28309 - 28332 (NC_045512)	FAM-ACC CCG CAT TAC GTT TGG TGG ACC-BHQ1
**Target 2**	SARS-CoV-2 Nucleocapsid; 29164 - 29183 (NC_045512)	TTA CAA ACA TTG GCC GCA AA
	SARS-CoV-2 Nucleocapsid; 29213 - 29230 (NC_045512)	GCG CGA CAT TCC GAA GAA
	SARS-CoV-2 Nucleocapsid; 29188 – 29210 (NC_045512)	FAM-ACA ATT TGC CCC CAG CGC TTC AG-BHQ1
**Target 3**	SARS-CoV-2 Envelope; 26269-26294 (NC_045512)	ACA GGT ACG TTA ATA GTT AAT AGC GT
	SARS-CoV-2 Envelope; 26381-26357 (NC_045512)	ATA TTG CAG CAG TAC GCA CAC A
	SARS-CoV-2 Envelope; 26332-26357 (NC_045512)	FAM-ACA CTA GCC ATC CTT ACT GCG CTT CG-BBQ
**Control**	Human RNase P; chr10:90872001-90872019 (hg38)	AGA TTT GGA CCT GCG AGC G
	Human RNase P; chr10:90872022-90872044 (hg38)	GAG CGG CTG TCT CCA CAA GT
	Human RNase P; chr10:90872046-90872065 (hg38)	FAM-TTC TGA CCT GAA GGC TCT GCG CG-BHQ1

Supplementary Table 7: The raw qPCR data of the six patient samples tested in Figure 2D. N represents 'not detected'.

Specimen number	qPCR				MORIARTY Slope	qPCR/MORIARTY COVID-19 result
	E	N1	N2	RNase P		
S1	14.36	15.23	16.13	23.66	213157.64	+/+
S2	17.16	15.12	16.48	23.79	205686.16	+/+
S3	15.59	15.39	16.52	26.54	174713.04	+/+
S4	30.09	28.07	29.76	28.38	146119	+/-
S5	29.23	26.19	27.91	26.17	124236	+/-
S6	N	N	N	25.45	4586	-/-

Supplementary Table 8: The raw qPCR data of the fourteen patients tested in Figure 3. N represents 'not detected'. I represents 'Invalidated'.

**Specimen number**	**qPCR**				**MORIARTY Slope**	**qPCR/MORIARTY COVID-19 result**
	**E**	**N1**	**N2**	**RNase P**		
S1	18.48	26.52	20.87	28.34	401740	+/+
S2	18.68	18.24	21.15	28.25	223191	+/+
S3	25.62	25.57	27.81	29.97	155311	+/+
S4	31.09	30.68	33.37	28.01	111717	+/+
S5	26.70	26.52	29.02	28.04	85790	+/+
S6	27.78	26.31	28.50	28.93	77486	+/+
S7	20.54	21.29	22.80	27.67	72924	+/+
S8	30.34	30.70	31.74	35.90	47212	+/+
S9	N	26.48	28.40	29.26	46864	+/+
S10	N	31.08	32.54	26.91	32498	+/-
S11	N	N	N	N	33707	I/-
S12	N	N	N	30.79	59078	-/+
S13	N	N	N	29.33	12402	-/-
S14	N	N	N	32.41	4586	-/-

Supplementary Figure 1, Related to Figures 1, 2, 3.

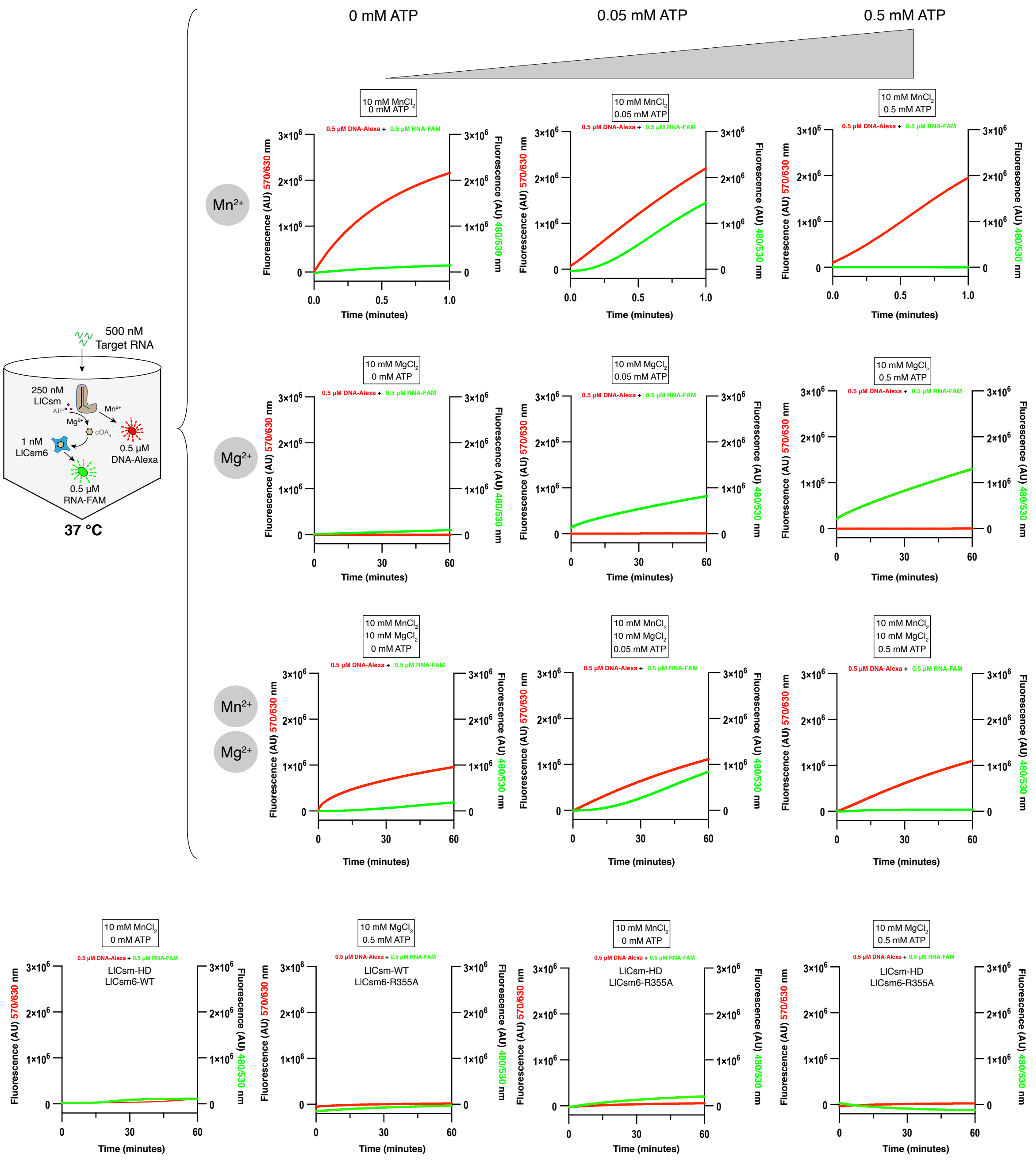

Supplementary Figure 1, Related to Figures 1, 2, 3. Construction of MORIARTY at different conditions. The schematics indicates components and their concentrations included in amplification-free MORIARTY reaction at 37 ºC. 500 nM model target RNA (Figure 1) was used as the stimulator. The requirement for the divalent ions for cOA_6 production and DNase is indicated. The RNase (via Csm6) and DNase activities are recorded by RNA-FAM (green) and DNA-Alexa (red) probes signal, respectively. All the curves are plotted using non-linear regression in GraphPad Prism and corrected for background using the signal stimulated with water. The combination of divalent ions and ATP concentrations is indicated for each trial reaction. Csm or Csm6 mutants tested at the corresponding metal ion conditions are labeled and shown at the last row.

Supplementary Figure 2, Related to Figure 2.

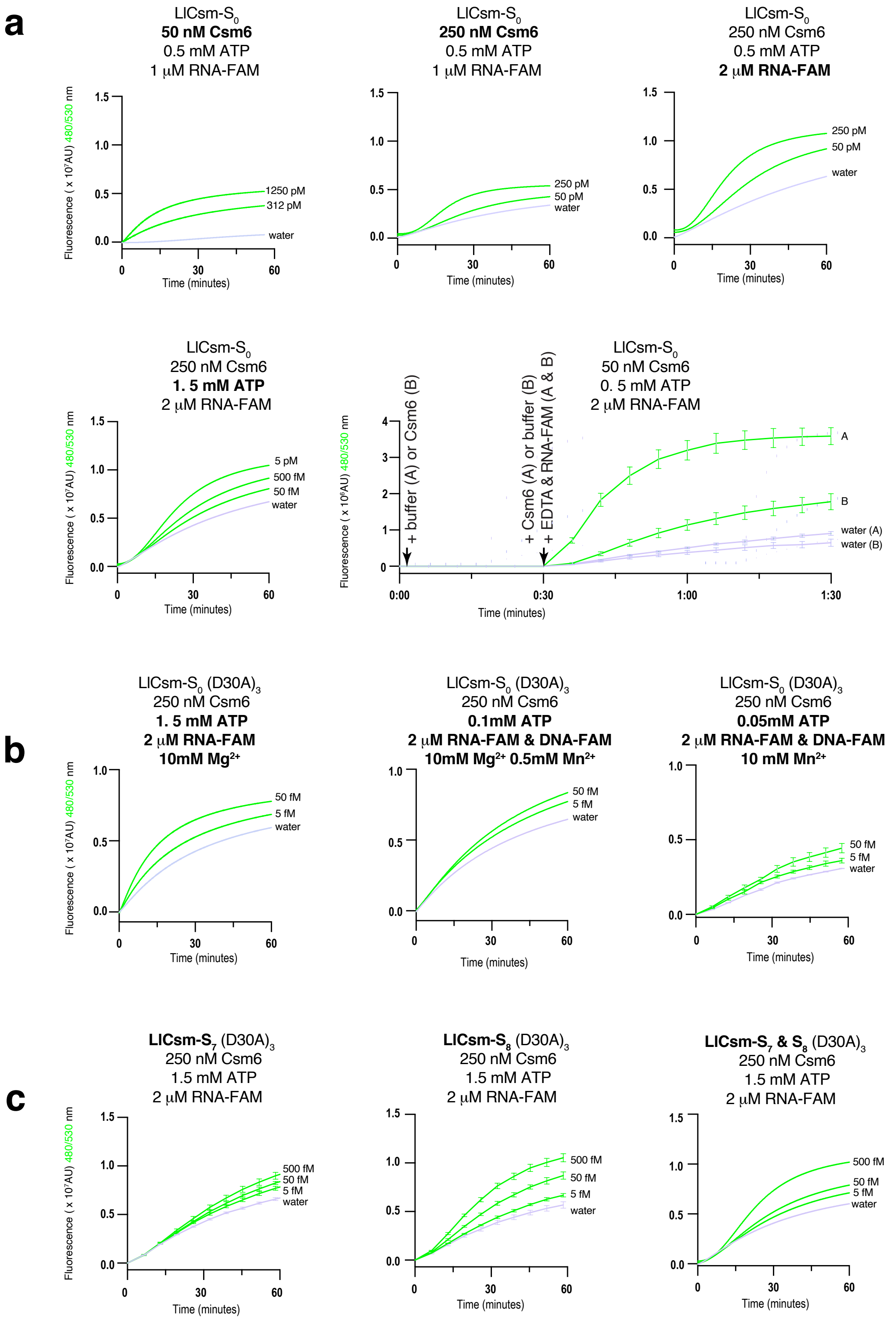

Supplementary Figure 2, Related to Figure 2. Optimization of amplification-free MORIARTY for three LlCsm effectors. LlCsm_S_0 targets SARS-CoV-2 S gene nucleotides 22280-22308, LlCsm_S_7 targets nucleotides 24702-24730 and LlCsm_S_8 targets nucleotides 25061-25089, respectively. The type and concentrations of the components are indicated for each panel with those varied in bold-faced font. All experiments used in vitro transcribed S mRNA (S_IVT_RNA) (green) or water (light purple) as the stimulator and Csm effector at 250 nM. (**a**) Variations of Csm6, ATP, RNA-FAM probe 10 mM $MgCl_2$ for low target RNA concentrations (50 fM – 1.25 nM) and with the wild-type LlCsm_S_0. All detection experiments were carried out for 60 minutes except for that of the Csm6 chase experiment was 90 minutes at 37 ºC. Triplicate of the Csm6 chase experiment was carried out and the error bars are indicated. (**b**) Compare the efficiency of the RNA cleavage-deficient LlCsm_S_0 $(D30A)_3$ (the Csm3 subunit containing D30A mutation) to that of wild type in A and the effects of different divalent ions. Triplicates were carried out for LlCsm_S_0 at the Mn^{2+} condition that can be compared with the triplicate of LlCsm_S_7 and LlCsm_S_8 in panel c. (**c**) Optimization of multiplexing with the three effectors at the Mg^{2+} condition against serially diluted S_IVT_RNA at 0.5 fM-500 fM concentrations. The LlCsm-S_7 and Llcsm-S_8 were tested individually in triplicates with error bars indicated and together.

Supplementary Figure 3, Related to Figure3.

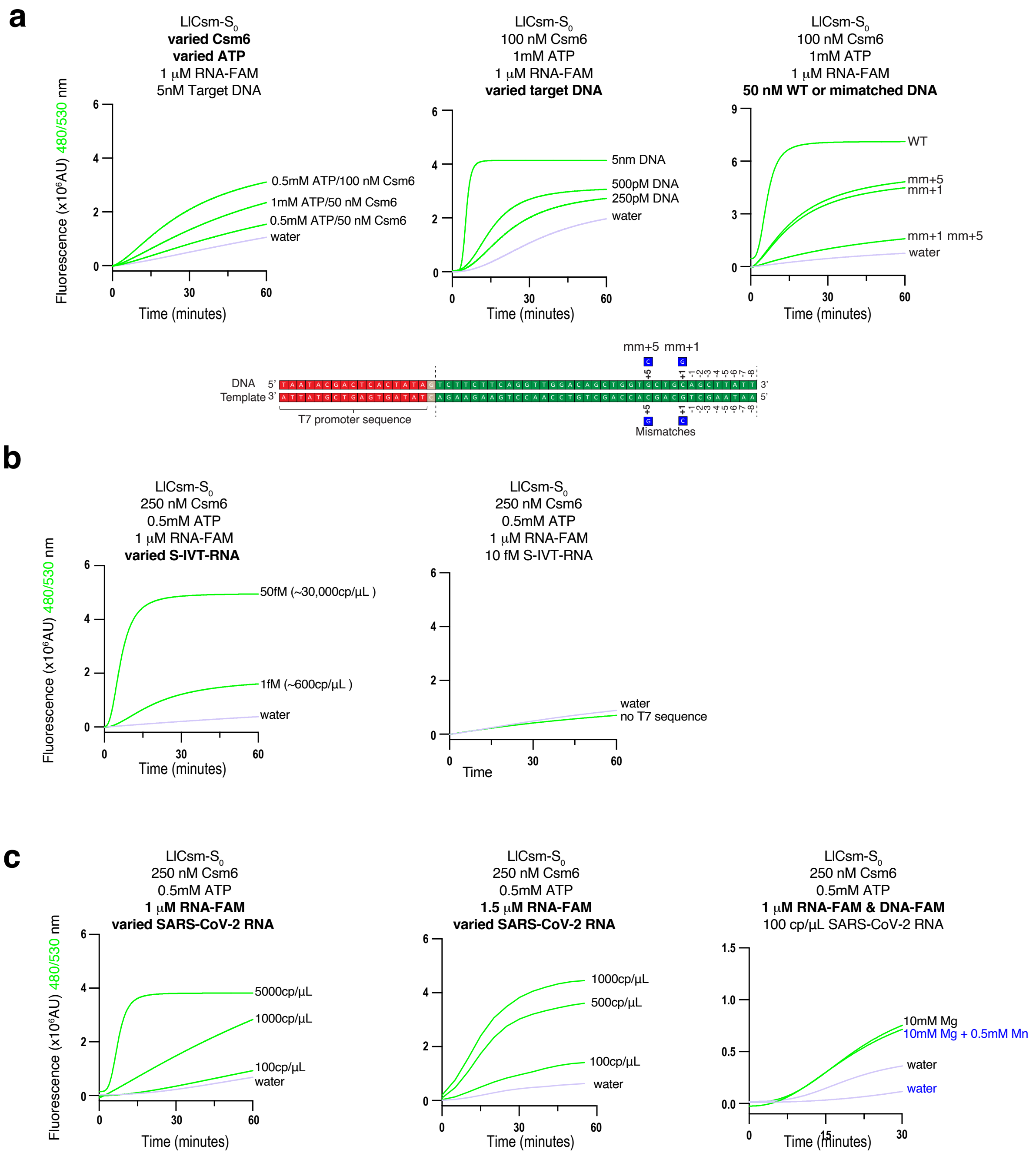

Supplementary Figure 3, Related to Figure3. Optimization of RT-RPA-T7 MORIARTY for the LlCsm_S_0 wild-type effector (250 nM). The type and concentrations of the components are indicated for each panel with those varied in bold-faced font. (**a**) Optimization of T7 MORIARTY without RT-RPA components with regards to Csm6, ATP, and DNA template concentrations. The DNA template used is shown below the panels and contains T7 promoter sequence fused to protospacer sequence encoding SARS-CoV-2 S gene complementary to S_0 crRNA or those contain mismatches at positions +1 or +5. The reaction conditions specific combinations of ATP and Csm6 (left) or the DNA concentrations (middle) are labeled. For testing sensitivity on mismatched DNA (right), either DNA containing single (mm+1 or mm+5) or both (mm+1 mm+5) were used as the stimulator. (**b**) Left, RT-RPA-T7 MORIARTY sensitivity to in vitro transcribed S mRNA (S_IVT_RNA) was estimated with the 100 μL RT-RPA reaction product from serially diluted S_IVT_RNA as the template. 2.5 μL RT-RPA product was added to the pre-mixed RT-RPA-T7 MORIARTY reaction. Right, the same reaction as that on the left with 10 fM S_IVT_RNA and RT-RPA primers minus the T7 promotor sequence (Supplementary Table 5). (**c**) RT-RPA-T7 MORIARTY sensitivity to independently quantified SARS-CoV2-RNA (BEI Resources) was estimated with the RT-RPA reaction products from serially diluted SARS-CoV2-RNA as the template that with water as the template. The effects of fluorescence probes and metal ions were tested.

Supplementary Figure 4, related to Figure 3.

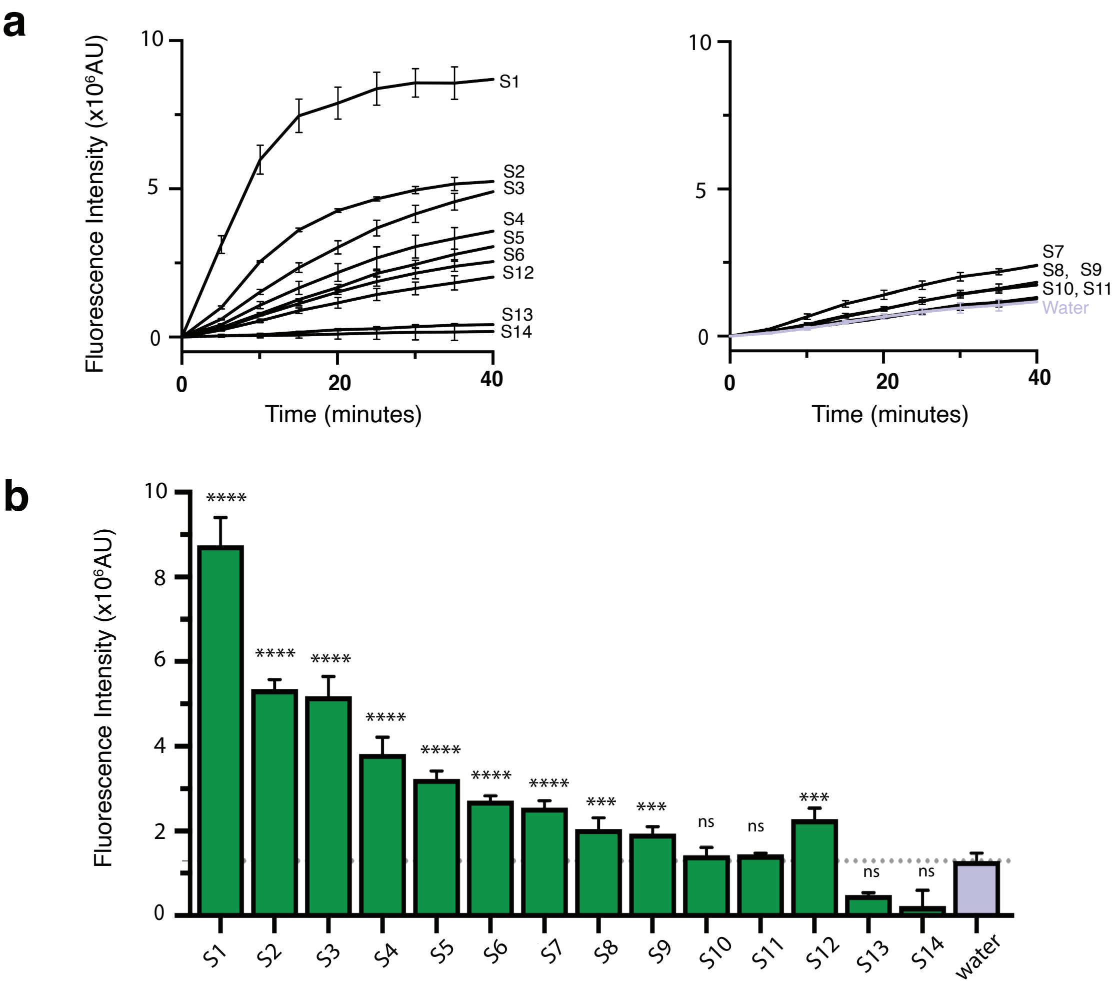

Supplementary Figure 4, related to Figure 3. Raw data of rise in fluorescence of patient samples tested in RT-RPA-T7-MORIARTY and two alternative analysis methods. (**a**) The two independent experiments testing two groups of the 14 patient samples (S1-S14). Each sample was tested in triplicate and error bars are indicated. (**b**) Bar diagram showing raw fluorescence intensities at 40 min time interval. The grey dotted line represents the intensity of water sample taken at the same time interval and defined as the negative threshold. Patient intensity data were compared to that of the negative control (water) by performing Ordinary one-way analysis of variance (ANOVA) and Dunnett's multiple comparisons test. **** denotes $p<0.0001$, *** denotes $p=0.0070$ and ns denotes non-significant.
