# Supplementary material for: Virus detection via programmable Type III-A CRISPR-Cas systems
Source: Nat Commun. 2021 Sep 27;12:5653. doi: 10.1038/s41467-021-25977-7 (PMC8476571; doi:10.1038/s41467-021-25977-7)
Supplement: Supplementary file 3 — Reporting Summary [file 41467_2021_25977_MOESM3_ESM.pdf]

nature research

Corresponding author(s): Hong Li

Last updated by author(s): Apr 21, 2021

Reporting Summary

Nature Research wishes to improve the reproducibility of the work that we publish. This form provides structure for consistency and transparency in reporting. For further information on Nature Research policies, see our Editorial Policies and the Editorial Policy Checklist.

Statistics

For all statistical analyses, confirm that the following items are present in the figure legend, table legend, main text, or Methods section.

n/a	Confirmed	
☐	☒	The exact sample size (*n*) for each experimental group/condition, given as a discrete number and unit of measurement
☐	☒	A statement on whether measurements were taken from distinct samples or whether the same sample was measured repeatedly
☐	☒	The statistical test(s) used AND whether they are one- or two-sided<br>*Only common tests should be described solely by name; describe more complex techniques in the Methods section.*
☒	☐	A description of all covariates tested
☒	☐	A description of any assumptions or corrections, such as tests of normality and adjustment for multiple comparisons
☒	☐	A full description of the statistical parameters including central tendency (e.g. means) or other basic estimates (e.g. regression coefficient) AND variation (e.g. standard deviation) or associated estimates of uncertainty (e.g. confidence intervals)
☐	☒	For null hypothesis testing, the test statistic (e.g. *F*, *t*, *r*) with confidence intervals, effect sizes, degrees of freedom and *P* value noted<br>*Give P values as exact values whenever suitable.*
☒	☐	For Bayesian analysis, information on the choice of priors and Markov chain Monte Carlo settings
☒	☐	For hierarchical and complex designs, identification of the appropriate level for tests and full reporting of outcomes
☒	☐	Estimates of effect sizes (e.g. Cohen's *d*, Pearson's *r*), indicating how they were calculated

Our web collection on statistics for biologists contains articles on many of the points above.

Software and code

Policy information about availability of computer code

Data collection	The fluorescence measurements were performed using Spectramax ID5 multi-mode microplate reader (Molecular Devices).
Data analysis	The fluorescence data acquisition was performed using SoftMax Pro 7 Software. The data analysis was performed using GraphPad Prism version 9. qPCR data was analyzed with 21 CFR Part 11 software module-enabled qPCRsoft384 software version 1.2.3.0.

For manuscripts utilizing custom algorithms or software that are central to the research but not yet described in published literature, software must be made available to editors and reviewers. We strongly encourage code deposition in a community repository (e.g. GitHub). See the Nature Research guidelines for submitting code & software for further information.

Data

Policy information about availability of data

All manuscripts must include a data availability statement. This statement should provide the following information, where applicable:

- Accession codes, unique identifiers, or web links for publicly available datasets
- A list of figures that have associated raw data
- A description of any restrictions on data availability

The SARS-CoV-2 sequence information was obtained from publicly available NCBI entry MT80105.1. The raw qPCR and T7-MORIARTY data related to Figures 2 & 3 is provided in Supplementary Tables 7 & 8. Raw fluorescence readings are provided as Source Data file.

April 2020

Field-specific reporting

Please select the one below that is the best fit for your research. If you are not sure, read the appropriate sections before making your selection.

[x] Life sciences [ ] Behavioural & social sciences [ ] Ecological, evolutionary & environmental sciences

For a reference copy of the document with all sections, see nature.com/documents/nr-reporting-summary-flat.pdf

Life sciences study design

All studies must disclose on these points even when the disclosure is negative.

Sample size	A total of 20 patient nasal swab samples were available to us for qPCR and MORIARTY-based SARS-CoV-2 diagnosis. Due to the sample volume limitation, triplicates were made for each patient sample. We found that this sample size and triplicates are sufficient as the variation among the replicates were small.
Data exclusions	All data associated with the 20 patients including their respective RNase P values, Ct values corresponding to SARS-CoV-2 E, N1, N2 genes and T7-MORIARTY slope values were used for data analysis. One patient sample was invalidated due to no RNase P value.
Replication	All fluorescence-based COVID-19 detection assays were successfully performed as triplicates and compared to water signals for consistency.
Randomization	All patients whose nasal swab samples were used for research in this study were randomized. There is no bias in sample collection.
Blinding	The qPCR and MORIARTY experiments were performed independently on the same set of nasal swab RNA extracts. Investigators were blind to group allocation during data collection or analysis.

Reporting for specific materials, systems and methods

We require information from authors about some types of materials, experimental systems and methods used in many studies. Here, indicate whether each material, system or method listed is relevant to your study. If you are not sure if a list item applies to your research, read the appropriate section before selecting a response.

Materials & experimental systems

n/a	Involved in the study	
[x]	[ ]	Antibodies
[x]	[ ]	Eukaryotic cell lines
[x]	[ ]	Palaeontology and archaeology
[x]	[ ]	Animals and other organisms
[ ]	[x]	Human research participants
[x]	[ ]	Clinical data
[x]	[ ]	Dual use research of concern

Methods

n/a	Involved in the study	
[x]	[ ]	ChIP-seq
[x]	[ ]	Flow cytometry
[x]	[ ]	MRI-based neuroimaging

Human research participants

Policy information about studies involving human research participants

Population characteristics	The nasal swab samples resuspended in the viral Universal Transport Media (UTM) used for SARS-CoV-2 diagnosis originated from Florida State University/Tallahassee Memorial Hospital COVID-19 testing center. Patient's age or gender or COVID-19 status at the time of sample collection were not known or restricted by the investigators. The associated patient data is protected in compliance with local regulations.
Recruitment	Samples were not collected through active recruiting patients.
Ethics oversight	Tallahassee Memorial Healthcare provides the Waiver of HIPAA authorization under 45 CFR 46.1.1(b) to this study (IRB #2021-52).

Note that full information on the approval of the study protocol must also be provided in the manuscript.
